# Multiparametric MR Index for the Diagnosis of Non-Alcoholic Steatohepatitis in Patients with Non-Alcoholic Fatty Liver Disease

**DOI:** 10.1038/s41598-020-59601-3

**Published:** 2020-02-14

**Authors:** Jeong Woo Kim, Young-Sun Lee, Yang Shin Park, Baek-Hui Kim, Soo Yeon Lee, Jong Eun Yeon, Chang Hee Lee

**Affiliations:** 1 Department of Radiology, Korea University Guro Hospital, Korea University College of Medicine, Seoul, Korea; 2 Department of Radiology, Korean Armed Forces Capital Hospital, Gyeonggi-do, Korea; 3 Department of Internal Medicine, Korea University Guro Hospital, Korea University College of Medicine, Seoul, Korea; 4 Department of Pathology, Korea University Guro Hospital, Korea University College of Medicine, Seoul, Korea

**Keywords:** Non-alcoholic fatty liver disease, Non-alcoholic steatohepatitis

## Abstract

Non-alcoholic steatohepatitis (NASH) is a complex disease consisting of various components including steatosis, lobular inflammation, and ballooning degeneration, with or without fibrosis. Therefore, it is difficult to diagnose NASH with only one imaging modality. This study was aimed to evaluate the feasibility of magnetic resonance imaging (MRI) for predicting NASH and to develop a non-invasive multiparametric MR index for the detection of NASH in non-alcoholic fatty liver disease (NAFLD) patients. This prospective study included 47 NAFLD patients who were scheduled to undergo or underwent ultrasound-guided liver biopsy within 2 months. Biopsy specimens were graded as NASH or non-NASH. All patients underwent non-enhanced MRI including MR spectroscopy (MRS), MR elastography (MRE), and T1 mapping. Diagnostic performances of MRS, MRE, and T1 mapping for grading steatosis, activity, and fibrosis were evaluated. A multiparametric MR index combining fat fraction (FF), liver stiffness (LS) value, and T1 relaxation time was developed using linear regression analysis. Receiver operating characteristic (ROC) curve analysis was performed to evaluate the diagnostic performance of the newly devised MR index. Twenty NASH patients and 27 non-NASH patients were included. Using MRS, MRE, and T1 mapping, the mean areas under the curve (AUCs) for grading steatosis, fibrosis, and activity were 0.870, 0.951, and 0.664, respectively. The multiparametric MR index was determined as 0.037 × FF (%) + 1.4 × LS value (kPa) + 0.004 × T1 relaxation time (msec) −3.819. ROC curve analysis of the MR index revealed an AUC of 0.883. The cut-off value of 6 had a sensitivity of 80.0% and specificity of 85.2%. The multiparametric MR index combining FF, LS value, and T1 relaxation time showed high diagnostic performance for detecting NASH in NAFLD patients.

## Introduction

Non-alcoholic fatty liver disease (NAFLD) is a leading cause of chronic liver disease with increasing prevalence worldwide^1^. NAFLD encompasses a wide spectrum of diseases, ranging from simple steatosis and non-alcoholic steatohepatitis (NASH) to liver cirrhosis. NASH increases the risk of hepatocellular carcinoma (HCC) and death from cardiovascular disease^2,3^. Therefore, it is crucial to differentiate NASH from simple steatosis. Liver biopsy is regarded as the gold standard for the diagnosis of NASH^4,5^. However, liver biopsy is an invasive and costly procedure with the risk of pain, bleeding, and although extremely rarely, even death. As a liver biopsy specimen represents only about 0.0002% of the whole liver, sampling error with inter- and intra-observer variability is another potential problem. Therefore, there have been substantial clinical demands for alternative and noninvasive methods to diagnose NASH.

With recent advances in MRI, MR spectroscopy (MRS), and MR elastography (MRE) have emerged as promising methods for detecting and grading fat and fibrosis, respectively^6–9^. MRS enables the direct measurement of the fat proton signal fraction and is considered the method of choice for accurate non-invasive quantification of liver fat^10^. MRE has shown promising results for staging hepatic fibrosis and detecting NASH in NAFLD patients^8,9^. It has also been shown that T1 mapping can be used to differentiate patients with liver fibrosis and cirrhosis^11^ and to predict clinical outcomes in patients with chronic liver disease^12^.

Although MRS, MRE, and T1 mapping have shown good diagnostic performance in detecting and grading NASH components such as steatosis or fibrosis, it is difficult to diagnose NASH with one modality only because NASH is characterized by various components including steatosis, lobular inflammation, ballooning degeneration, and fibrosis. Therefore, we postulated that a non-invasive multiparametric MR index combining MRS, MRE, and T1 mapping may help diagnose NASH in NAFLD patients, thereby potentially reducing the need for liver biopsy.

The purpose of this study was to evaluate the feasibility of MRI for predicting NASH and to develop a non-invasive multiparametric MR index for the detection of NASH in NAFLD patients.

## Results

Patients were classified into the NASH (n = 20) or non-NASH (n = 27) group based on the Steatosis, Activity, Fibrosis (SAF) scoring system. Mean age was significantly higher in the NASH group than in the non-NASH group (p = 0.007). Fasting glucose level was significantly higher and platelet count was significantly lower in the NASH group than in the non-NASH group (p = 0.012 and 0.001, respectively). There was no significant difference in other clinical and laboratory data between these two groups (Table 1). Based on histopathologic evaluation, steatosis was graded as S0 (n = 0), S1 (n = 25), S2 (n = 18), or S3 (n = 4); hepatocyte ballooning as B0 (n = 27), B1 (n = 10), or B2 (n = 10); lobular inflammation as L0 (n = 0), L1 (n = 15), or L2 (n = 32); and fibrosis as F0 (n = 14), F1 (n = 13), F2 (n = 12), F3 (n = 7), or F4 (n = 1).

**Table 1:** Baseline characteristics of patients.

	Total patients (n = 47)	Non-NASH (n = 27)	NASH (n = 20)	p-value
Age (years)	51.0 ± 12.7	46.9 ± 12.7	56.6 ± 10.4	0.007
Male: Female	16:31	11:16	5:15	0.477
Body mass index (kg/m^2^)	28.3 ± 6.2	27.9 ± 7.7	28.7 ± 3.7	0.612
ALT (IU/L)	80.2 ± 43.1	82.2 ± 53.0	81.0 ± 28.4	0.926
AST (IU/L)	59.6 ± 26.5	55.8 ± 30.2	67.3 ± 18.8	0.134
ALP (IU/L)	88.2 ± 21.3	90.7 ± 24.4	86.1 ± 17.9	0.474
GGT (IU/L)	79.0 ± 61.1	95.0 ± 76.2	62.9 ± 30.9	0.059
Total bilirubin (mg/dL)	0.6 ± 0.3	0.6 ± 0.3	0.6 ± 0.3	0.986
Total cholesterol (mg/dL)	181.9 ± 36.4	190.1 ± 33.1	173.8 ± 40.0	0.133
Triglycerides (mg/dL)	154.9 ± 65.3	168.2 ± 68.9	144.8 ± 59.1	0.224
HDL-cholesterol (mg/dL)	43.5 ± 11.1	44.2 ± 11.1	42.8 ± 11.9	0.674
LDL-cholesterol (mg/dL)	112.6 ± 33.2	118.0 ± 34.0	106.3 ± 33.5	0.247
Fasting glucose (mg/dL)	117.0 ± 32.4	105.9 ± 16.3	133.7 ± 41.9	0.012
Albumin (g/dL)	4.2 ± 0.6	4.1 ± 0.8	4.2 ± 0.3	0.692
Platelet count ( × 10^3^/L)	207.8 ± 54.1	228.0 ± 46.8	177.5 ± 49.9	0.001

Note. – ALT alanine aminotransferase, AST aspartate aminotransferase, ALP alkaline phosphatase, GGT γ-glutamyltransferase, HDL high density lipoprotein, LDL low density lipoprotein.

### MR parameters and histopathologic components

Diagnostic performance of MRS for grading steatosis is summarized in Table 2. Using MRS, the mean area under the curve (AUC) for grading steatosis was 0.870. Diagnostic performances of MRE and T1 mapping for grading activity (ballooning and lobular inflammation) and fibrosis are summarized in Table 2. Using MRE, the mean AUCs for grading activity (ballooning and lobular inflammation) and fibrosis were 0.825 and 0.951, respectively. Using T1 mapping, the mean AUCs for grading activity and fibrosis were 0.664 and 0.615, respectively.

**Table 2:** Diagnostic performance of MRS, MRE and T1 mapping for grading each histopathologic component.

			Mean AUC	AUC	Cut-off value*	Sensitivity (%)	Specificity (%)
MRS	Steatosis	≥S2	0.870	0.862	12.88	81.8	92.0
		≥S3		0.878	19.08	100.0	81.4
MRE	Ballooning^†^	≥B1	0.825^†^	0.898	3.31	90.0	81.5
		≥B2		0.811	3.47	90.0	67.6
	Lobular inflammation^†^	≥L2		0.765	3.13	65.6	86.7
	Fibrosis	≥F1	0.951	0.991	2.58	97.0	100.0
		≥F2		0.879	3.13	97.0	100.0
		≥F3		0.984	4.34	100.0	92.3
T1 mapping	Ballooning^†^	≥B1	0.664^†^	0.624	843.29	95.4	60.7
		≥B2		0.682	921.57	90.0	56.4
	Lobular inflammation^†^	≥L2		0.686	894.98	71.9	64.7
	Fibrosis	≥F1	0.615	0.614	1064.37	34.3	92.9
		≥F2		0.659	921.57	69.6	61.5
		≥F3		0.572	894.678	88.9	45.0

*Units of MRS, MRE, and T1 mapping are percentage (%), kilopascal (kPa), and millisecond (msec), respectively.

^†^The mean values of AUCs for grading activity (ballooning and lobular inflammation) are demonstrated.

In subgroup analysis, the mean T1 value was significantly higher in group B (mean FF_MRS_ > 15%) than in group A (mean FF_MRS_ ≤ 15%) for ≥ F2 (significant fibrosis) patients (1133.9 ± 97.0 vs. 935.9 ± 79.6 msec, p = 0.001). AUCs of T1 mapping for grading ≥ F2 (significant fibrosis) were 0.837 and 0.714 in group A and B, respectively. These results indicated that hepatic fat is a confounding factor of T1 value estimation.

### MR index

**Figure 1:**
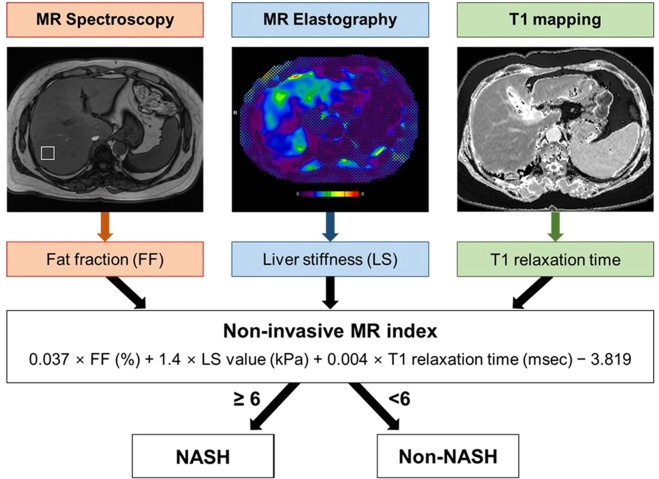
Development of a non-invasive multiparametric MR index. Using linear regression analysis, a non-invasive multiparametric MR index combining fat fraction (FF), liver stiffness (LS) value, and T1 relaxation time measured on MRS, MRE, and T1 mapping was determined as 0.037 × FF (%) + 1.4 × LS value (kPa) + 0.004 × T1 relaxation time (msec) − 3.819.

**Figure 2:**
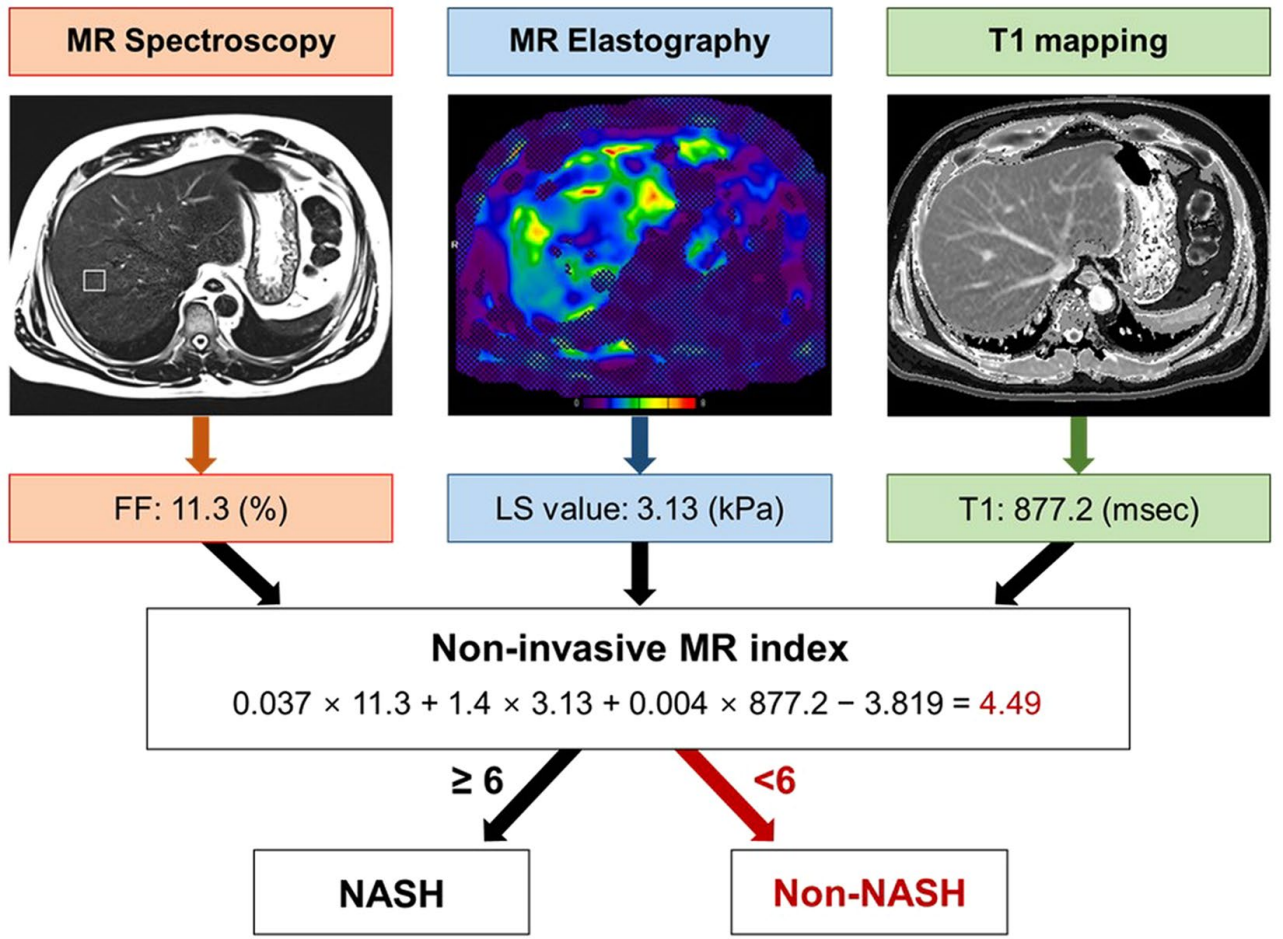
An example of using the MR index to predict NASH. A 51-year-old man with clinically suspected NASH who underwent percutaneous liver biopsy and MR imaging. Fat fraction measured on MR spectroscopy was 11.3%, liver stiffness value measured on MR elastography was 3.13 kPa, and T1 relaxation time measured on T1 mapping was 877.2 msec. The non-invasive multiparametric MR index predicted that the patient was a non-NASH patient. Histopathologic analyses of the patient’s biopsy specimens revealed grade 1 steatosis, grade 0 balloon degeneration, grade 1 lobular inflammation, and grade 1 fibrosis. Therefore, he was classified as a non-NASH patient according to the SAF scoring system.

A multiparametric MR index combining fat fraction (FF) (measured on MRS), liver stiffness (LS) value (on MRE) and T1 relaxation time (on T1 mapping) was determined as 0.037 × FF (%) + 1.4 × LS value (kPa) + 0.004 × T1 relaxation time (msec) −3.819 (Fig. 1). Receiver operating characteristic (ROC) curve analysis revealed an AUC of 0.883 and an optimal cut-off value of 4.6 with a corresponding sensitivity of 95.0% and specificity of 77.8% (Table 3). The specificity was relatively low whereas the sensitivity was high. To exclude patients who do not need a liver biopsy, because specificity is more important than the sensitivity, a cut-off value of 6 exhibiting a relatively high specificity while maintaining the sensitivity was used (sensitivity of 80.0% and specificity of 85.2%) (Table 3, Fig. 2).

**Table 3:** Diagnostic performance of multiparametric MR index.

	AUC	Cut-off value	Sensitivity (%)	Specificity (%)
Entire group	0.883	4.6	95.0	77.8
		6.0	80.0	85.2
Group A (mean FF_MRS_ ≤ 15%)	0.909	4.3	100.0	83.3
Group B (mean FF_MRS_ > 15%)	0.901	4.6	100.0	66.7

In subgroup analysis, the MR index showed higher diagnostic performances in both group A and B than in the entire group. In group A (mean FF_MRS_ ≤ 15%), the MR index showed an AUC of 0.909 and an optimal cut-off value of 4.3 with a corresponding sensitivity of 100% and specificity of 83.3%. In group B (mean FF_MRS_ > 15%), the MR index showed an AUC of 0.901 and an optimal cut-off value of 4.6 with a corresponding sensitivity of 100.0% and specificity of 66.7% (Table 3).

## Discussion

In our study, MRS and MRE showed high diagnostic performance for staging steatosis (mean AUC 0.870) and fibrosis (mean AUC 0.951) in NAFLD patients, respectively. These results are in good agreement with those of previous studies in which MRS and MRE showed strong correlations with hepatic FF and hepatic fibrosis, respectively^8,13–15^. Although MRS and MRE are considered good modalities for diagnosing and grading steatosis and fibrosis, respectively, it is difficult to diagnose NASH with one modality only because NASH is a complex disease consisting of various components including steatosis, lobular inflammation, and ballooning degeneration, with or without fibrosis. Therefore, we devised a new multiparametric MR index combining FF, LS value, and T1 relaxation time. Our newly devised MR index predicted NASH in NAFLD patients with a sensitivity of 80.0% and a specificity of 85.2%.

NAFLD is a term used to describe fat accumulation in the liver with no history of excess alcohol intake or other liver disease. NASH is a subgroup of NAFLD, characterized by hepatocellular injury, inflammation, and ballooning with or without fibrosis. There are several staging and grading systems for NASH. The most widely used system is the NASH Activity Score (NAS), which was originally developed as a tool for evaluating therapeutic outcomes in NAFLD patient trials. It has proven useful for comparative or interventional studies. However, it is less beneficial as a diagnostic tool for NASH and has low prognostic value because it does not include fibrosis^16^. The more recently developed SAF scoring system may be more accurate for identifying NASH and be more relevant for long-term prognostication^17,18^.

Hepatic fat is an essential component for diagnosing NAFLD/NASH. There have been several efforts to identify hepatic steatosis using various imaging techniques including ultrasonography^19^, unenhanced computed tomography^20,21^, MRI^22^, and MRS^23,24^. MRS is considered the most precise non-invasive imaging technique for hepatic fat quantification. In the past, MRS was performed with free-breathing, resulting in long acquisition times and erroneous data acquisition due to motion artifacts^25^. Recently, high-speed T2-corrected multi-echo (HISTO) MRS was introduced for T2-corrected hepatic fat measurement with a single breath hold^26,27^. In our study, MRS showed high mean AUC of 0.870 for grading steatosis in NAFLD patients.

NAFLD ranges from simple steatosis to NASH, which can progress to cirrhosis and HCC. Although liver biopsy is considered the only reliable method for distinguishing NASH from simple steatosis, there is the potential risk of sampling error and complications such as pain, bleeding, and although extremely rarely, even death. These drawbacks of liver biopsy have increased the demand for alternative and noninvasive methods for diagnosis of NASH. These include non-invasive models composed of demographic and laboratory parameters, such as aspartate aminotransferase to platelet ratio index (APRI), fibrosis-4 index (FIB-4), BARD score, and NAFLD fibrosis score (NFS)^18^. Non-invasive imaging techniques such as acoustic radiation force impulse (ARFI), transient elastography (FibroScan), shear wave elastography (SWE), and MRE have also been studied to detect fibrosis in NAFLD patients^18^. Among them, MRE showed the highest diagnostic accuracy for the grading and diagnosis of fibrosis in NAFLD patients in meta-analysis and prospective studies^28,29^. In our study, MRE showed high mean AUC of 0.951 for grading fibrosis in NAFLD patients.

T1 mapping was originally used for non-invasive evaluation of myocardial fibrosis, which is characterized by edema (increase in tissue water) and increased extracellular matrix (ECM) remodeling (increase in collagen volume fraction) after an acute ischemic event^30,31^. Several studies have assessed this technique in chronic liver disease patients^11,32,33^. In liver fibrosis, increased edema and ECM remodeling occur after hepatocyte injury, resulting in lengthened T1 relaxation time. The T1 mapping technique directly measures T1 relaxation time in milliseconds. Banerjee *et al*. demonstrated that T1 relaxation time was strongly correlated with liver fibrosis in 79 patients who underwent liver biopsy^11^. T1 mapping had an AUC of 0.94 with a sensitivity of 86% and a specificity of 93% for differentiating healthy people and patients with no fibrosis from those with fibrosis. In our study, compared with MRE, T1 mapping showed relatively low diagnostic performances for grading fibrosis (mean AUC, 0.615 vs. 0.951) and activity (mean AUC, 0.664 vs. 0.825). This result may be due to the fact that T1 values are influenced by hepatic fat^34^. Therefore, subgroup analyses were performed to evaluate the diagnostic performance of T1 mapping for grading significant fibrosis (F2) and the diagnostic performance of MR index for detecting NASH. Diagnostic performance of T1 mapping improved in each subgroup (FF_MRS_ ≤ 15% and > 15%) relative to the entire group. In particular, diagnostic performance was higher in group A (FF_MRS_ ≤ 15%) than in group B (FF_MRS_ > 15%). Diagnostic performance of the MR index also improved in each group relative to the entire group and was higher in group A than in group B. These results indicate that hepatic fat is a confounding factor of the T1 value, therefore the T1 value should be used with caution in patients with fatty liver of moderate or greater degree. Applying different MR indices according to hepatic fat component can overcome the limitation of T1 mapping. To the best of our knowledge, there is no new sequence yet to improve T1 mapping.

As noted above, MRI studies in NAFLD/NASH have focused primarily on the diagnosis of fat or fibrosis, which are components of NASH. Like non-invasive models using clinical data, if a non-invasive MR index is developed by combining MR parameters in NAFLD patients, it would provide an easily available prediction model in the clinical setting. Therefore, a new MR index was devised by combining FF, LS value, and T1 relaxation time measured by MRS, MRE, and T1 mapping, which are included in our MRI protocol for NAFLD patients. This new MR index predicted NASH as a SAF score with high diagnostic performance.

This study had several limitations. First, only a few patients limited to a single tertiary center using one MRI scanner were included. Although a small cross-vendor validation study comparing the reproducibility of two difference MR scanners from Philips and GE Healthcare in 13 subjects demonstrated that LS value measurements were reproducible and had good consistency across two vendors^35^, a multicenter study with a larger number of patients using several different MRI scanners is needed to develop an MR index that can be widely used in various clinical settings. Second, our new devised MR index was not validated using a validation group due to the small number of patients. Another prospective study using a validation group of NAFLD patients is needed to validate the results of this study. Third, it is well known that hepatic iron deposition can be increased associated with liver disease^36^, and age, gender, and menopause for female may affect hepatic iron deposition^37,38^, which in turn may affect MRI measurements. However, it was not possible to perform subgroup analyses according to age, gender, and menopause due to small number of patients. We hope to study the effect of hepatic iron deposition on MRI measurements in the future with a larger number of patients. Fourth, in our study, a non-invasive index was developed using only MR parameters and linear regression analysis with three fixed MR parameters (FF, LS value, and T1 relaxation time). However, the aim of the study was to develop a simple MR index for predicting NASH in NAFLD patients. In the future, it is expected to devise a NASH index through a prospective study using multivariate regression analysis of clinical (demographic and laboratory) data and MR parameters.

In conclusion, MRI showed high diagnostic performance for detecting and grading the NASH components of steatosis, activity (lobular inflammation and ballooning degeneration), and fibrosis. Our newly devised MR index combining MRS, MRE, and T1 mapping also demonstrated high diagnostic performance for detecting NASH in NAFLD patients. Therefore, this multiparametric MR index may help diagnose NASH in NAFLD patients and potentially reduce the need for liver biopsy.

## Materials and Methods

### Study design

This clinical trial was a prospective study conducted in accordance with the Declaration of Helsinki at the Korea University Guro Hospital in Korea. The study was approved by the Institutional Review Board of the Korea University Guro Hospial (approval no.: KUGH16184) and written informed consent was obtained from each patient. This trial was registered into ClinicalTrials.gov on 22 November 2017. The study followed CONSORT guidelines in the reporting of results.

### Patients

Patients with clinically suspected NASH who were scheduled to undergo or underwent liver biopsy within 2 months were identified from October 2016 to June 2017. Patients 18 years and older who voluntarily participated in this study were included. Exclusion criteria were as follows: (a) other known causes of chronic liver disease such as chronic hepatitis B or C, autoimmune hepatitis, and primary sclerosing cholangitis; (b) use of steatogenic medications within the past six months; (c) significant alcohol consumption (more than 70 g per week for women and 140 g per week for men); (d) history of HCC; (e) pregnancy; and (f) contraindications to perform MRI. Finally, 47 patients were included in this study. Demographic and clinical data including age, gender, height, weight, and body mass index were obtained. Laboratory data within 1 month of the MR exam were also recorded. These are summarized in Table 1.

### Histopathological evaluation

All patients underwent percutaneous liver biopsies and at least two cores of liver tissue were obtained from the right hepatic lobe by using the Tru-cut technique with an 18-gauge automatic needle device (TSK Laboratory, Tochigi, Japan). An experienced pathologist with more than 15 years of experience, who was blinded to the patients’ clinical and radiologic data, reviewed all liver biopsy specimens. Biopsy specimens were graded according to the SAF scoring system as NASH (steatosis score > = 1, lobular inflammation > = 1, ballooning > = 1, and any fibrosis score) or non-NASH. The SAF score is a semi-quantitative score of steatosis (0–3), activity [lobular inflammation (0–2) + ballooning (0–2)], and fibrosis (0–4). Steatosis score (S) was assessed according to the amount of large or medium-sized lipid droplets without foamy microvesicles, ranging from 0 to 3 (S0, <5%; S1, 5–33%; S2, 34–66%; S3, >67%). Activity score (A) is the unweighted sum of hepatocyte ballooning (0–2) and lobular inflammation (0–2), ranging from 0 to 4. Hepatocyte ballooning was graded as B0, none; B1, few balloon cells; or B2, many cells/ prominent ballooning. Lobular inflammation was graded as L0, none; L1, <2 foci/ 200 fields; or L2; ≥2 foci/ 200 fields. Fibrosis score was evaluated using the NASH-Clinical Research Network scoring system as follows: F0, no fibrosis; F1, perisinusoidal fibrosis in zone 3 (stage 1a or 1b) or portal/periportal fibrosis (stage 1c); F2, perisinusoidal and portal/periportal fibrosis; F3, bridging fibrosis; and F4, cirrhosis.

### MR examination

All patients underwent MR imaging with a 3 T MR scanner (MAGNETOM Skyra, Siemens Healthineers, Erlangen, Germany). T2-weighted half-Fourier acquisition single-shot turbo spin-echo (HASTE) sequence, 3-dimensional T1-weighted gradient-recalled echo volumetric interpolated breath-hold examination (VIBE) sequence, MR spectroscopy, MR elastography, and T1 mapping were acquired.

#### MR spectroscopy

**Figure 3:**
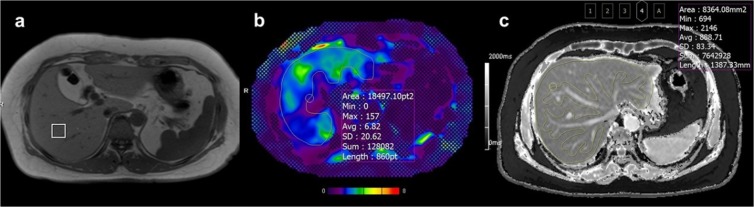
Measurement of MR parameters using regions-of-interest (ROIs). (**a**) A square-shaped voxel on MR spectroscopy (**b**) A free-hand ROI on the stiffness map of MR elastography **(c)** A free-hand ROI on T1 mapping.

Single-voxel MRS was performed using a prototypical HISTO MR spectroscopic technique. The HISTO technique is a modified stimulated echo acquisition mode (STEAM) sequence^27^. MRS spectra were obtained as the same method described in our previously published study^27^. A single voxel (20 × 20 × 20 mm) was placed in the right hepatic posterior segment by an experienced technologist (more than 10 years of experience in MRS) while avoiding large bile ducts, vessels, and focal hepatic lesions (Fig. 3a). Parameters included repetition time (TR), 3000 msec; mixing time, 10 msec; and 5 echo times (TEs) of 12, 24, 36, 48, and 72 msec. Each MRS acquisition was performed within 15 seconds during one breath hold. This process was repeated three times. Post-processing of the MRS data was performed using inline software of the MR vendor.

#### MR elastography

A pneumatic driver system (Resoundant, Rochester, MN, USA) was used to induce shear waves into the liver. A cylindrical passive driver was placed against the patient’s right anterior chest wall at the level of the xiphoid process^8^. Continuous acoustic vibrations of 60 Hz were transmitted from the active driver to the passive driver through a flexible vinyl tube^8^. Measurement parameters of a prototypical MR elastography 2D spin-echo based echo-planar sequence were as follows: TR/TE, 100/47 msec; SPAIR fat suppression; field of view (FOV), 380 mm × 380 mm; matrix size, 100 × 100; slice thickness, 6 mm; and interslice gap, 1.2 mm. Four MR elastography sections were obtained in each patient and it took 11 seconds to obtain four slices. Patients were requested to hold their breath at the end of expiration. After the acquisition was completed, wave images were processed inline by the MR scanner to generate stiffness maps (elastograms) in kilopascals (kPa). Elastograms were displayed with a scale corresponding to the stiffness value.

#### T1 mapping

For T1 mapping, a prototypical 2-dimensional Look-Locker inversion recovery T1 mapping sequence based on fast low-angle shot (FLASH) with inline construction was performed. Three axial slices were obtained at the level of confluence of the hepatic veins to the inferior vena cava, portal hilum, and gallbladder fossa within a breath hold. For each slice the Look-Locker T1 mapping sequence applies a non-selective inversion recovery pulse followed by low-flip angle FLASH acquisitions of multiple inversion contrasts. Imaging parameters were as follows: TR/TE, 3.0/1.32 msec; FA, 8°; number of excitations, 1.0; FOV, 380 × 309 mm; matrix size, 192 × 154; acceleration factor, 2; slice thickness, 8 mm; number of inversion contrasts, 16.

### Image analysis

#### Measurement of fat fraction on MRS

Liver FFs were calculated automatically and displayed as a percentage (%). Mean FF values were used as representative values.

#### Measurement of liver stiffness on MRE

To measure liver stiffness (LS) values, two board-certified radiologists (C.H.L. and J.W.K. with 25 years and 6 years of experience in abdominal radiology, respectively), blinded to the clinical data and pathologic results, drew free-hand regions-of-interest (ROIs) along the liver margin by consensus, avoiding large vessels, liver edges, and motion artifacts. ROIs were manually drawn on wave images where wave propagations were regular and relatively free of reflections and interference patterns. ROIs drawn on wave images were copied and pasted onto the stiffness maps (Fig. 3b). One ROI per slice (total 4 slices) was measured, and mean LS values (in kilopascals) were used as representative values.

#### Measurement of T1 relaxation time on T1 mapping

To measure T1 relaxation time, the same two radiologists, blinded to the clinical data and pathologic results, drew free-hand ROIs along the liver margin on each slice of the T1 maps by consensus, avoiding major intrahepatic vessels and focal hepatic lesions (Fig. 3c). One ROI per slice (total 3 slices) was measured, and mean T1 relaxation times (in milliseconds) were used as representative values.

### Statistical analysis

For clinical and laboratory data, the independent-sample t-test was used to compare continuous variables, and the chi-square (χ^2^) test was used to compare categorical variables between the NASH and non-NASH groups.

To evaluate the diagnostic performances of MRS, MRE, and T1 mapping for grading each histopathologic component (steatosis, ballooning, locular inflammation, and fibrosis), ROC curve analyses were performed and AUCs were obtained.

A previous study demonstrated that the T1 value estimates may be affected by liver fat^34^. Therefore, subgroup analyses were performed to evaluate the diagnostic performances of T1 mapping and the MR index using ROC curve analyses. Patients were assigned to one of two groups based on mean FF_MRS_: group A (mean FF_MRS_ ≤ 15%) and group B (mean FF_MRS_ > 15%).

A p-value < 0.05 indicated a statistically significant difference. All statistical analyses were performed using statistical software (SPSS version 20.0 for Windows, SPSS, Chicago, IL; and MedCalc for Windows, version 9.3.2.0, MedCalc Software, Mariakerke, Belgium).

### Development of the MR index

A multiparametric MR index as a weighted linear combination of liver signal FF, LS value, and T1 relaxation time was developed using linear regression analysis to predict the SAF score (Fig. 1). ROC curve analysis was performed to evaluate the diagnostic performance of the MR index.
